# A New Method for the Calculation of Characteristics of Disc Springs with Trapezoidal Cross-Sections and Rounded Edges

**DOI:** 10.3390/ma15051954

**Published:** 2022-03-06

**Authors:** Dominik Sebastian Leininger, Max Benedikt Geilen, Marcus Klein, Matthias Oechsner

**Affiliations:** Center for Engineering Materials (MPA-IfW), Technical University of Darmstadt, Grafenstraße 2, 64283 Darmstadt, Germany; max.geilen@tu-darmstadt.de (M.B.G.); marcus.klein@tu-darmstadt.de (M.K.); matthias.oechsner@tu-darmstadt.de (M.O.)

**Keywords:** disc springs, Belleville washers, characteristics, idealized geometry, cross-section, non-rectangular, trapezoidal, rounded edges

## Abstract

In the European standards specifying disc spring manufacturing, geometry, shape and characteristic, an edge rounding is prescribed. Common methods for the calculation of disc spring characteristics, even in these standards, are based on a rectangular cross-section. This discrepancy can lead to a considerable divergence of the computed characteristic from the characteristic determined by testing. In literature, this divergence has not yet been examined with regard to rounded edges. In this paper, a new method addressing this problem is introduced. For this purpose, the geometry of idealized disc springs is parameterized. Based on four edge radii and two angles of the inner and outer faces, equations to compute the initial cone angle and the lever arm are introduced. These equations are used to formulate an algorithm to adapt other computation methods to non-rectangular cross-sections and rounded edges. The method is applied to the formulas by Almen–Laszlo, Curti–Orlando, Zheng and those by Kobelev. FE simulations of disc springs with rounded edges and a non-rectangular cross-section were used to verify the new formulas. The results show that the introduced method can be applied to known characteristic computation methods and result in a model expansion taking cross-section variations into account. The adjusted characteristics show more accurate alignment to the FE simulation for the cross-section variations investigated. These findings not only close the geometric gap between the manufacturing guidelines and the computation on an analytical basis, they also define a new parameter space for designs of disc springs and a corresponding force computation method to optimize spring characteristics.

## 1. Introduction

Disc springs are axially loaded conical shaped washers with a wide range of applications [1,2,3,4,5,6,7,8,9,10,11,12,13,14,15,16,17,18,19,20,21,22,23,24,25,26,27,28,29,30,31,32,33,34]. They are specified in the European standards DIN EN 16983 [35] and DIN EN 16984 [36]. The theoretical geometry of a spring is defined with a rectangular cross-section and sharp edges, whose characteristics can be calculated with formulas from Section 5 in DIN EN 16984 [36]. However, the manufacturing process defined by Table 7 in DIN EN 16983 [35] includes edge rounding.

As prescribed in Section 1 in DIN EN 16983 [35], proper function of a disc spring includes compliance with the spring force tolerances, among other requirements. Despite the development of different analytical approaches to compute force—deflection relations, the simplifications made in the geometry can cause inaccuracies in the calculation of disc spring characteristics. Since the manufacturing process can also lead to a non-rectangular, i.e., trapezoidally-skewed, cross-section, in addition to the radii, this non-conformance is also to be considered. Therefore, in this paper the idealized geometry of such disc springs is described and a new computational approach is introduced to take the geometric variations into account. The described method can be combined with different existing computation methods for disc springs with rectangular cross-section and sharp edges.

A finite element (FE) simulation was used in order to be able to evaluate the consequences of a wide range of different geometries. For setup and preprocessing of the FE models, the Spring_stack-module [37,38] was used.

The models used employ first order, reduced integration, axisymmertic finite elements sized approximately one percent of the spring thickness. Contact plates are implemented as analytical rigids. They are connected to the springs through a frictionsless, hard, surface to surface, finite sliding contact. The characteristics are computed by continuously increasing the displacement of the contact place close to edge I.

Literature on the characteristics of disc springs dates back to 1936 when Almen and Laszlo published their well-known formula [39]. The standard noted above also uses a modified version of this computational method. Almen and Laszlo used the tangential stresses to link the load of a disc spring with its deflection. Curti and Orlando published a similar approach incorporating radial stresses [40]. An energy-based approach to compute the characteristics was published by Zheng et al. [41]. Koblev [42] presents an analytical approach capable of approximating high working angle disc springs. Luxemburg and Givli [43] model non-rigid rotation.

Different approaches consider friction through an additional frictional moment [44], through a master curve concept [45] or through a coupling of axial and radial forces [42]. Some approaches allow the combination of different frictional coefficients [46,47,48] and rate-dependent frictional coefficients [47,48]. The latter [47,48] also considers contact stiffness.

Several models have been generated to model deviating geometries, including slotted disc springs [44,49,50,51,52], variable thickness disc springs [53,54,55] and disc springs with contact flats [56,57].

As an alternative to analytical approaches, disc springs have been modelled using FE simulations [2,3,4,5,8,10,13,30,58,59,60,61,62,63,64,65,66,67,68,69,70,71,72,73,74,75,76,77,78,79,80,81,82,83]. FE simulations are computationally more expensive than analytical approaches. Furthermore, they often depend on software licenses. The main advantages, however, are the easy variation in geometry and the simulation of different material properties.

## 2. Idealized Geometry of Disc Springs

**Figure 1:**
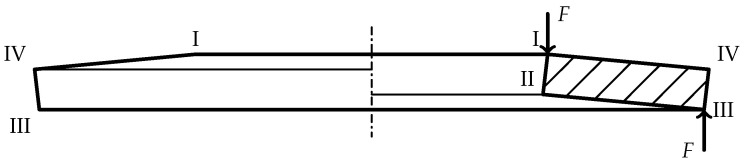
Half-section of a disc spring with rectangular cross-section with sharp edges.

In the standards DIN EN 16983 [35] and DIN EN 16984 [36], the disc spring and its cross-section are characterized by the four edges I through IV as shown in Figure 1. The loading force *F* acts on edges I and III .

**Figure 2:**
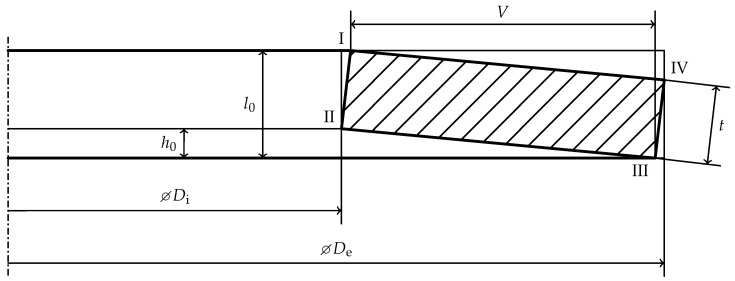
Dimensioned right half of a disc spring section.

The dimensions of the spring are measured with the inner and outer diameter, $D_{i}$ and $D_{e}$ , the total height $l_{0}$ and the thickness *t* [35,36] as shown in Figure 2. The lever arm *V* measures the radial distance of the force application edges. Note that DIN EN 16983 [35] and DIN EN 16984 [36] the depiction of the lever arm *V* is different than in Figure 1. It is shown as the radial distance between the points I and IV , not between I and III , Figure 1a in [35,36]. This may be a consequence of the last revision correcting the depiction of $D_{e}$ , which shares the same extension line with the dimension of *V*. The vertical space below edge II is characterized by the cone height $h_{0}$ . It is approximated by

(1) $$\begin{array}{c} h_{0,\mathrm{DIN}}=l_{0}-t \end{array}$$

using the small angle approximation [35,36]. The cone height is used to compute the deflection to flat position, $s_{f}$ , in which the upper and lower faces are horizontal in an idealized model.

**Figure 3:**
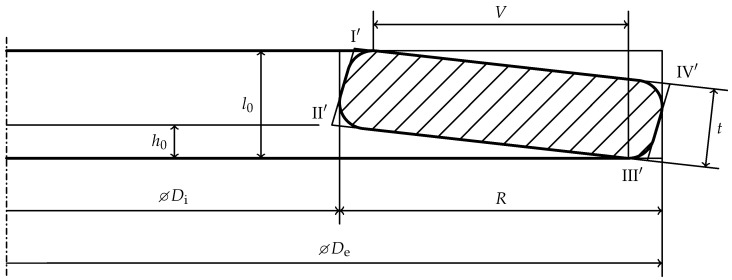
Dimensioned right half of a disc spring section with non-rectangular cross-section and rounded edges.

Figure 3 shows an idealized cross-section of an exemplary real disc spring. The virtual position of the sharp edges are named $I^{\prime }\;$ through ${\mathrm{IV}}^{\prime }\;$ , as, due to edge rounding, they are not material edges. The lever arm *V* in Figure 3 differs from the one in Figure 2. Furthermore, the deflection $s_{f}$ to flat position can not be directly measured as the cone height $h_{0}$ between ${\mathrm{II}}^{\prime }\;$ and the lower force application point, which moves on the circular arc of edge III during loading. The deflection $s_{f}$ to flat position is defined as

(2) $$\begin{array}{c} s_{f}=l_{0}-t \end{array}$$

which is generally not identical to the cone height $h_{0}$ , but matches the approximated cone height $h_{0,\mathrm{DIN}}$ for rectangular cross-sections with sharp edges.

**Figure 4:**
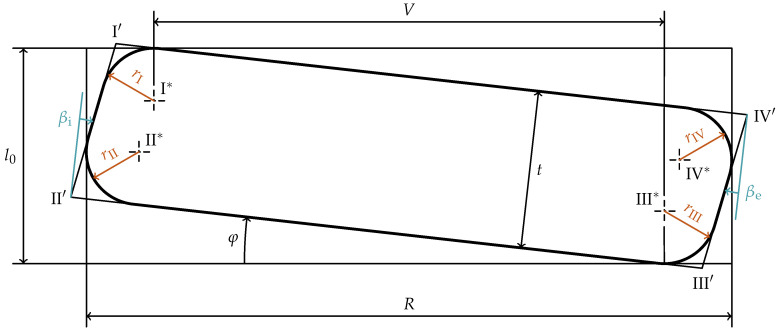
Definition of an idealized cross-section of a real disc spring ( $\beta_{i},\beta_{e}<0$ ).

The non-rectangularity of the cross-section is idealized by a trapezoid with rounded corners described by the parameters shown in Figure 4. The four radii $r_{I}$ through $r_{\mathrm{IV}}$ characterize the rounding of the corresponding edges with the center points $I^{*}\;$ through ${\mathrm{IV}}^{*}\;$ . The trapezoidality caused by the inner and outer face of the disc spring is measured counterclockwise on the right cross-section of the spring by the angles $\beta_{i}$ and $\beta_{e}$ . Thus, positive values of these angles cause the inner and outer faces to align towards the axial direction of the disc spring. The upper and lower faces are assumed to be parallel.

Since the load is applied axially, the lever arm *V* (equal to the radial distance of the force application lines) can be measured as the radial distance of the points $I^{*}\;$ and ${\mathrm{III}}^{*}\;$ . The initial slope angle of the disc spring is named φ .

Due to the fact that in the following investigations only geometric quantities of the cross-section are considered and thus the distance between the cross-section and the center-line of the disc spring can be neglected, the complexity is reduced by introducing the difference in radius *R*:

(3) $$\begin{array}{c} R=\frac{D_{e}-D_{i}}{2} \end{array}$$

**Figure 5:**
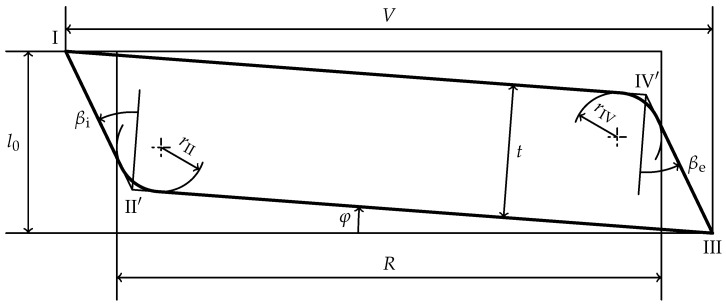
Definition of the geometric quantities for $\beta_{i},\beta_{e}>\varphi$ .

The dimensions *R* and $l_{0}$ are measured as shown in Figure 4, so the points $I^{\prime }\;$ through ${\mathrm{IV}}^{\prime }\;$ lie outside of the rectangle described by these quantities. Thus, these values are measurable for real disc springs using a caliper. While this is only true for $\beta_{i},\beta_{e}>\varphi$ , such large angles of the inner and outer faces are fairly uncommon in practice. In Figure 5 a cross-section with $\beta_{i},\beta_{e}>\varphi$ is shown. Note that the difference in radius *R* is measured from the arcs defining the edge rounding of the same edges as in Figure 4, II and IV , even though the points to measure are inside the cross-section and do not mark the radial extent of the cross-section in this case.

## 3. Calculation of Initial Slope Angle φ and Lever Arm V

**Figure 6:**
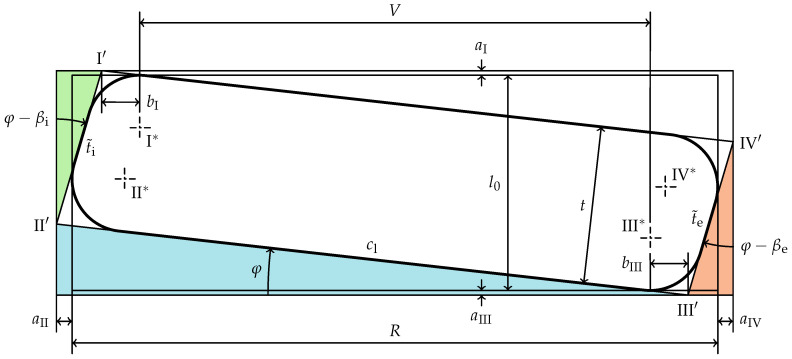
Cross-section with bounding boxes to calculate the initial slope angle φ and the lever arm *V* ( $\beta_{i},\beta_{e}<0$ ).

As shown in Figure 6, the bounding box of the rounded off cross-section with dimensions *R* and $l_{0}$ can be enlarged to fit the cross-section without edge rounding. This outer bounding box touches the points $I^{\prime }\;$ through ${\mathrm{IV}}^{\prime }\;$ and its offset is described by the four parameters $a_{I}$ through $a_{\mathrm{IV}}$ which are added to $l_{0}$ and *R*, respectively.

The width of the lower face $c_{l}$ as well as the height of the outer and inner face, ${\tilde{t}}_{i}$ and ${\tilde{t}}_{e}$ , can be calculated to:

(4) $$\begin{array}{ccc} c_{l} & = & \left|\bar{\;{\mathrm{II}}^{\prime }\;\;\;{\mathrm{III}}^{\prime }\;\;\;}\right| \end{array}$$

(5) $$\begin{array}{ccccc} {\tilde{t}}_{i} & = & \left|\bar{\;I^{\prime }\;\;\;{\mathrm{II}}^{\prime }\;\;\;}\right| & = & \frac{t}{\cos(\beta_{i})} \end{array}$$

(6) $$\begin{array}{ccccc} {\tilde{t}}_{e} & = & \left|\bar{\;{\mathrm{III}}^{\prime }\;\;\;{\mathrm{IV}}^{\prime }\;\;\;}\right| & = & \frac{t}{\cos(\beta_{e})} \end{array}$$

Thus, ${\tilde{t}}_{e}$ and ${\tilde{t}}_{i}$ are always greater *t* as long as the angles $\beta_{i}$ and $\beta_{e}$ are not equal to zero and their absolute values are in an ordinary range, e.g., $\left|\beta_{i}\right|,\left|\beta_{e}\right|<45{}^{\circ}$ .

Therefore, the radial width of the outer bounding box can be calculated using the blue and the orange triangle in Figure 6:

(7) $$\begin{array}{c} a_{\mathrm{II}}+R+a_{\mathrm{IV}}=\cos\left(\varphi \right)\;c_{l}+\sin\left(\varphi -\beta_{e}\right)\;{\tilde{t}}_{e} \end{array}$$

Analogously, the blue and green triangles describe the axial height of the cross-section’s outer bounding box:

(8) $$\begin{array}{c} a_{I}+l_{0}+a_{\mathrm{III}}=\sin\left(\varphi \right)\;c_{l}+\cos\left(\varphi -\beta_{i}\right)\;{\tilde{t}}_{i} \end{array}$$

Eliminating $c_{l}$ , from Equations (7) and (8) follows:

(9) $$\begin{array}{c} \frac{a_{\mathrm{II}}+R+a_{\mathrm{IV}}-\sin\left(\varphi -\beta_{e}\right)\;{\tilde{t}}_{e}}{\cos(\varphi )}=\frac{a_{I}+l_{0}+a_{\mathrm{III}}-\cos\left(\varphi -\beta_{i}\right)\;{\tilde{t}}_{i}}{\sin(\varphi )} \end{array}$$

Thus, the initial slope angle φ can be calculated as the fixed point of the following equation:

(10) $$\begin{array}{cc} \varphi & =\arctan\left(\;\frac{a_{I}+l_{0}+a_{\mathrm{III}}-\cos\left(\varphi -\beta_{i}\right)\;{\tilde{t}}_{i}}{a_{\mathrm{II}}+R+a_{\mathrm{IV}}-\sin\left(\varphi -\beta_{e}\right)\;{\tilde{t}}_{e}}\;\right) \end{array}$$

For known initial slope angle φ , the lever arm *V* can be calculated as follows. Similar to Equation (7), another formula including *V* can be used to calculate the radial distance of the points ${\mathrm{II}}^{\prime }\;$ and ${\mathrm{IV}}^{\prime }\;$ by using the orange and green triangles in Figure 6:

(11) $$\begin{array}{c} a_{\mathrm{II}}+R+a_{\mathrm{IV}}=\sin\left(\varphi -\beta_{i}\right)\;{\tilde{t}}_{i}+b_{I}+V+b_{\mathrm{III}}+\sin\left(\varphi -\beta_{e}\right)\;{\tilde{t}}_{e} \end{array}$$

(12) $$\begin{array}{c} V=R+a_{\mathrm{II}}+a_{\mathrm{IV}}-b_{I}-b_{\mathrm{III}}-\sin\left(\varphi -\beta_{i}\right)\;{\tilde{t}}_{i}-\sin\left(\varphi -\beta_{e}\right)\;{\tilde{t}}_{e} \end{array}$$

To determine the unknown lengths $a_{I}$ through $a_{\mathrm{IV}}$ as well as the radial distance $b_{I}$ of the points $I^{*}\;$ , $I^{\prime }\;$ and the radial distance $b_{\mathrm{III}}$ of the points ${\mathrm{III}}^{*}\;$ , ${\mathrm{III}}^{\prime }\;$ , the rounded edges are examined. Exemplarily, edge I is discussed below.

**Figure 7:**
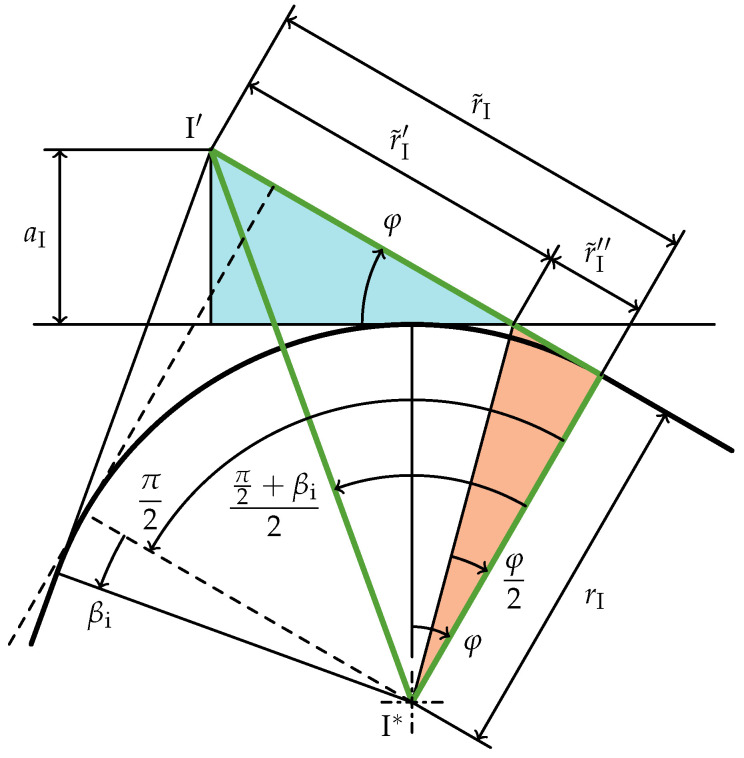
Geometry of edge I to calculate $a_{I}$ ( $\beta_{i}>0$ ). The dashed lines show the geometry with $\beta_{i}=0$ .

As shown in Figure 7, the distance $a_{I}$ measures the protrusion of $I^{\prime }\;$ out of the inner bounding box which touches the rounded cross-section. It follows from the geometry of the blue triangle in Figure 7 that:

(13) $$\begin{array}{cc} a_{I} & =\sin\left(\varphi \right)\;{\tilde{r}}_{I}^{\prime } \end{array}$$

where ${\tilde{r}}_{I}^{\prime }$ can be calculated using the orange triangle in Figure 7.

(14) $$\begin{array}{cccc} & {\tilde{r}}_{I}^{\prime } & & ={\tilde{r}}_{I}-{\tilde{r}}_{I}^{\prime \prime } \end{array}$$

(15) $$\begin{array}{cccc} & {\tilde{r}}_{I}^{\prime \prime } & & =\tan\left(\frac{\varphi }{2}\right)r_{I} \end{array}$$

As depicted in Figure 7, ${\tilde{r}}_{I}$ is computed as the tangent of the bisecting angle of the sum of the right angle of a rectangular cross-section (dashed in Figure 7) combined with the deviation from that, $\beta_{i}$ , visualized by the green-stroked triangle in Figure 7:

(16) $$\begin{array}{cc} {\tilde{r}}_{I} & =\tan\left(\frac{\frac{\pi }{2}+\beta_{i}}{2}\right)r_{I} \end{array}$$

Inserting ${\tilde{r}}_{I}$ from Equation (14) into Equation (13) by using Equations (16) and (15), yields

(17) $$\begin{array}{cc} a_{I} & =\sin\left(\varphi \right)\left(\tan\left(\frac{\frac{\pi }{2}+\beta_{i}}{2}\right)-\tan\left(\frac{\varphi }{2}\right)\right)r_{I} \end{array}$$

**Figure 8:**
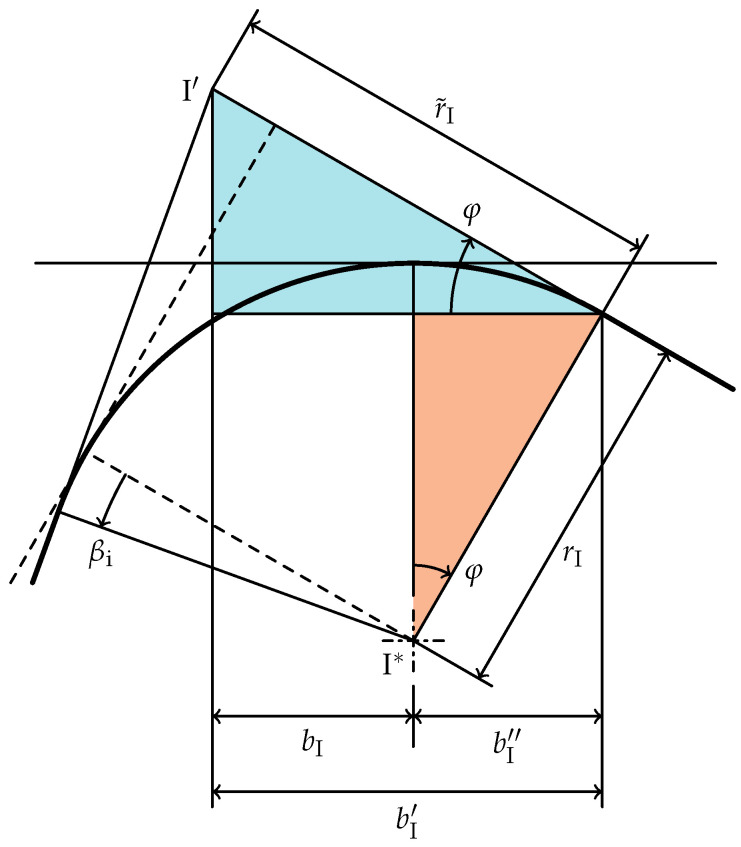
Geometry of edge I to calculate $b_{I}$ ( $\beta_{i}>0$ ). The dashed lines show the geometry with $\beta_{i}=0$ .

To calculate $b_{I}$ (necessary for *V*), the already known ${\tilde{r}}_{I}$ from Equation (16) is used. As Figure 8 shows, $b_{I}$ can be described as the difference between $b_{I}^{\prime }$ and $b_{I}^{\prime \prime }$ , which in turn can be computed using the blue and orange triangles in Figure 8:

(18) $$b_{I}=b_{I}^{\prime }-b_{I}^{\prime \prime }$$

(19) $$\begin{array}{ccc} b_{I}^{\prime } & = & \cos\left(\varphi \right)\;{\tilde{r}}_{I} \end{array}$$

(20) $$\begin{array}{ccc} b_{I}^{\prime \prime } & = & \sin\left(\varphi \right)\;r_{I} \end{array}$$

This leads to the following equation to calculate $b_{I}$ :

(21) $$\begin{array}{cc} b_{I} & =\left(\cos\left(\varphi \right)\;\tan\left(\frac{\frac{\pi }{2}+\beta_{i}}{2}\right)-\sin\left(\varphi \right)\right)r_{I} \end{array}$$

(22) $$\begin{array}{cc} \; & =\cos\left(\varphi \right)\left(\tan\left(\frac{\frac{\pi }{2}+\beta_{i}}{2}\right)-\tan\left(\varphi \right)\right)r_{I} \end{array}$$

The derivations of the parameters of the other edges, $a_{\mathrm{II}}$ through $a_{\mathrm{IV}}$ and $b_{\mathrm{II}}$ through $b_{\mathrm{IV}}$ , are outlined in Appendix A.

## 4. Calculation of Deformed Geometries of Loaded Disc Springs

In the previous sections, the disc spring was always assumed to experience neither deflection nor deformation. Under load, these assumptions obviously can’t hold true. In this section, a rotated cross-section of a deflected disc spring will be analyzed to overcome these restrictions. The cross-section is considered to be undistortable but able to rotate. This simplification is considered to be a good approximation and is used in several other computation approaches [39,40].

Since the beginning of Section 3, the cross-section is considered to be independent of the centerline of the disc spring. Since the loading does not affect this assumption, all measures are relative to the cross-section and the cross-section is assumed to be undistortable, the rotation center can be chosen freely, as the translation with respect to another rotation center, e.g., the neutral point in [39], is irrelevant. Here, the center of rotation is chosen as fixed at the center of edge rounding ${\mathrm{III}}^{*}\;$ . This leads to a fixed position of ${\mathrm{III}}^{*}\;$ during deflection and only the other point in question, the center of edge rounding $I^{*}\;$ , experiences displacement under load.

**Figure 9:**
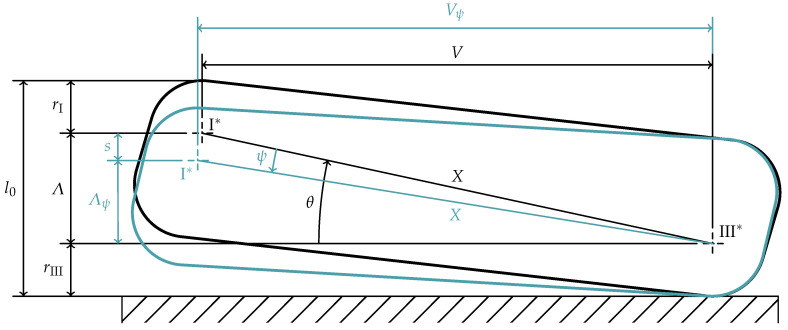
Cross-section of a unloaded (black) and loaded (blue) disc spring.

In Figure 9, the rotated cross-section is shown in blue after a rotation by ψ . This rotation results in a deflection *s* and an slightly lengthened lever arm $V_{\psi }$ . The total height $l_{0}$ of an unloaded (black) disc spring can be split up by introducing the axial distance Λ between the points $I^{*}\;$ and ${\mathrm{III}}^{*}\;$ , as shown in Figure 9:

(23) $$\begin{array}{cc} \Lambda & =l_{0}-r_{I}-r_{\mathrm{III}}, \end{array}$$

The radial distance between $I^{*}\;$ and ${\mathrm{III}}^{*}\;$ is measured with the already known lever arm *V*.

The angle θ , describing the angle between the horizontal and the connection line of the two points $I^{*}\;$ and ${\mathrm{III}}^{*}\;$ , as well as their distance *X* can be computed as:

(24) $$\begin{array}{cc} \theta & =\arctan\left(\frac{\Lambda }{V}\right) \end{array}$$

(25) $$\begin{array}{cc} X & =\sqrt{\Lambda^{2}+V^{2}} \end{array}$$

With these quantities, it follows from the definition of sine and cosine that:

(26) $$\begin{array}{cccc} & V & = & \cos(\theta )\;X \end{array}$$

(27) $$\begin{array}{ccc} \Lambda & = & \sin(\theta )\;X \end{array}$$

They can be generalized for the loaded disc spring by subtracting the rotation angle ψ from the initial value θ :

(28) $$\begin{array}{cc} V_{\psi } & =\cos(\theta -\psi )\;X \end{array}$$

(29) $$\begin{array}{ccccccc} & & & & & & =\sin(\psi )\;\Lambda +\cos(\psi )\;V \end{array}$$

(30) $$\begin{array}{cc} \Lambda_{\psi } & =\sin(\theta -\psi )\;X \end{array}$$

Furthermore, the following equation can be obtained from Figure 9 to describe the deflection *s* of the disc spring.

(31) $$\begin{array}{cc} s & =\Lambda -\Lambda_{\psi } \end{array}$$

By using Equations (27) and (30), the following equation gives the axial deflection *s* as a function of the rotation angle ψ :

(32) $$\begin{array}{cc} s & =\left(\sin\left(\theta \right)-\sin\left(\theta -\psi \right)\right)\;X \end{array}$$

The inverse of this,

(33) $$\begin{array}{cc} \psi & =\theta -\arcsin\left(\sin\left(\theta \right)-\frac{s}{X}\right)\;, \end{array}$$

is used in Equation (28) to calculate the deflection-dependent lever arm $V_{\psi }$ as a function of the deflection *s*:

(34) $$\begin{array}{cc} V_{\psi } & =\cos\left(\arcsin\left(\sin\left(\theta \right)-\frac{s}{X}\right)\right)X \end{array}$$

(35) $$\begin{array}{cc} \; & =\sqrt{1-{\left(\sin\left(\theta \right)-\frac{s}{X}\right)}^{\;2}}\;X \end{array}$$

By setting ψ=φ the disc spring is in flat position and the deflection holds the condition $s=s_{f}$ . With Equation (32), $s_{f}$ can be calculated as follows:

(36) $$\begin{array}{cc} s_{f} & =\left(\sin(\theta )-\sin(\theta -\varphi )\right)X \end{array}$$

It can be shown that this is equivalent to Equation (2) for rectangular cross-sections with sharp edges.

It can be shown that $s_{f}$ is generally unequal to the cone height $h_{0}$ of point ${\mathrm{II}}^{\prime }\;$ above the lower force application point of the spring. This is due to the non-rectangularity and the rounded edges of the cross-section. The different definition of $h_{0}$ in the standard, which uses the small angle approximation and define the cone height $h_{0,\mathrm{DIN}}$ as in Equation (1), Table 1 in [36], may cause an additional divergence of the two measures for non-rectangular cross-section and rounded edges.

The reduced height $l_{\psi }$ of a loaded disc spring can be computed analogous to Equation (23) as

(37) $$\begin{array}{cc} l_{\psi } & =r_{I}+\Lambda_{\psi }+r_{\mathrm{III}}. \end{array}$$

For a disc spring compressed to flat position ( $s=s_{f}$ , ψ=φ ), $l_{\psi }$ is equal to *t*.

Most analytical methods approximate the lever arm as the deflection-independent difference in radius *R*. It can be shown that for cross-sections satisfying condition (38), this leads to an slight overestimation of the lever arm and thus to a slight underestimation of the force.

(38) $$\begin{array}{cc} R-V_{\psi }> & 0\Leftrightarrow \\ R\left(1-\cos(\psi )\right)> & \left(l_{0}-r_{I}-r_{\mathrm{III}}\right)\;\sin\left(\psi \right)\\ & +\left(a_{\mathrm{II}}+a_{\mathrm{IV}}-b_{I}-b_{\mathrm{III}}-\sin\left(\varphi -\beta_{i}\right)\times {\tilde{t}}_{i}-\sin\left(\varphi -\beta_{e}\right)\times {\tilde{t}}_{e}\right)\cos\left(\psi \right) \end{array}$$

## 5. Calculation of Spring Characteristics

In this section the basic approach to adjust the calculation of characteristics to non-rectangular cross-sections with rounded edges is presented. Subsequently, this is used to adapt the algorithm of Almen–Laszlo to those geometries as an example.

### 5.1. Concept

In the original formulas, the necessary force to create a specific deflection *s* is computed based on a set of basic geometry parameters of a rectangular cross-section with sharp edges described in Section 2. The algorithm adjustment described below is based on the use of a more precise lever arm and two geometric modifications to increase the accuracy of the original formulas when applied to non-rectangular cross-sections and rounded edges: the first shape modification adjusts the diameters and the total height to values as they would be of a rectangular cross-section with sharp edges. This leads to an increase of the area of the cross-section, which must be compensated by the second modification. These two modifications result in the description of a cross-section compatible with the original formulas with properties very similar to those of a spring with a non-rectangular cross-section and rounded edges under investigation.

As dicussed in Section 3 and Section 4, due to the rounded edges and the non-rectangularity of the cross-section, the lever arm of such springs can differ considerably from the one with a rectangular cross-section and sharp edges. This is accounted for by using the deflection-dependent lever arm $V_{\psi }$ described in Equation (34) instead of using e.g., the deflected independent difference in radius *R*, as Almen and Laszlo do to approximate the lever arm.

**Figure 10:**
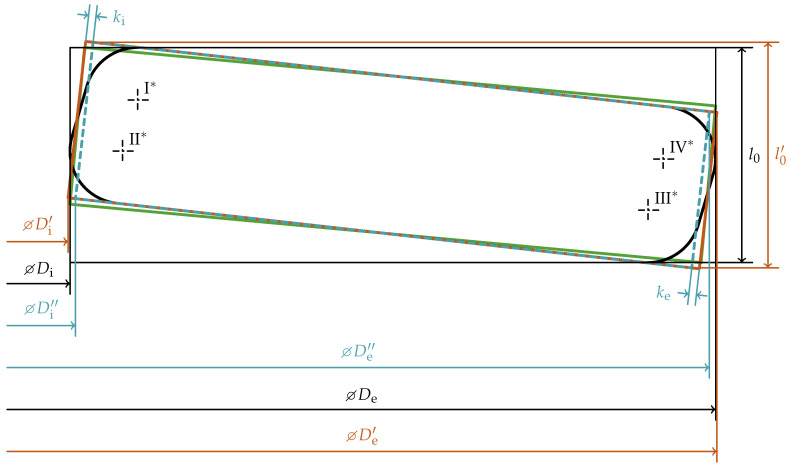
Comparison of an idealized cross-section (black, $\beta_{i},\beta_{e}<0$ ), a rectangular cross-section based on the basic geometry parameters (green, same *R* and $l_{0}$ as black but a rectangular cross-section and sharp edges, the cross-section area and the slope angle differs) and a rectangular cross-section (orange) to have similar properties as the idealized cross-section (black). The dashed blue lines show the cross-section after the second shape adjustment with the corrections $k_{i}$ and $k_{e}$ to keep the cross-section area approximately the same. Its slope angle is the same as the orange and the black one’s.

As the original computation methods are designed for rectangular cross-sections with sharp edges, the shape modifications noted above are used to imitate this kind of cross-section, while retaining the basic characteristics such as the initial slope angle. Figure 10 shows a comparison of the different cross-sections.

It can be observed that the diameters $D_{e}$ and $D_{i}$ as well as the total height $l_{0}$ need to be slightly modified to the adjusted diameters $D_{e}^{\prime }$ , $D_{i}^{\prime }$ and the adjusted total height $l_{0}^{\prime }$ to maintain the basic properties of the disc spring such as the initial slope angle. The outer diameter $D_{e}$ is increased by $a_{\mathrm{II}}$ on both sides of the disc spring to imitate sharp edges. Furthermore, a negative angle $\beta_{e}$ of the outer face of the disc spring (as shown in Figure 10) causes the outer diameter to be larger, which is compensated on both sides of the disc spring (factor 2) by the last term in the equation below:

(39) $$\begin{array}{cc} D_{e}^{\prime } & =D_{e}+2a_{\mathrm{II}}-2\times \cos\left(\varphi \right)\tan\left(-\beta_{e}\right)\;\frac{t}{2}\\ \; & =D_{e}+2a_{\mathrm{II}}-\cos\left(\varphi \right)\tan\left(-\beta_{e}\right)\;t \end{array}$$

Analogously, the following equation is obtained for the adjusted inner diameter $D_{i}^{\prime }$ :

(40) $$\begin{array}{cc} D_{i}^{\prime } & =D_{i}-2a_{\mathrm{IV}}+\cos\left(\varphi \right)\tan\left(-\beta_{i}\right)\;t \end{array}$$

The total height $l_{0}$ must be adjusted by $a_{I}$ and $a_{\mathrm{III}}$ to compensate the edge rounding and the influence of the angles $\beta_{i}$ and $\beta_{e}$ .

(41) $$\begin{array}{cc} l_{0}^{\prime }= & l_{0}+a_{I}+a_{\mathrm{III}}-\sin\left(\varphi \right)\left(\tan\left(-\beta_{i}\right)+\tan\left(-\beta_{e}\right)\right)\;\frac{t}{2} \end{array}$$

Thus, the above adjustments create a cross-section with the edge radii, outer face angle and inner face angle of zero, but retaining the slope angle and the length of the cross-section’s center-line. With this, the cross-section area is slightly larger which results in an increased resistance to deformation. This is compensated by the second adjustment of the diameter measures which result in $D_{e}^{\prime \prime }$ and $D_{i}^{\prime \prime }$ :

(42) $$\begin{array}{cc} D_{e}^{\prime \prime } & =D_{e}^{\prime }-2k_{e} \end{array}$$

(43) $$\begin{array}{cc} D_{i}^{\prime \prime } & =D_{i}^{\prime }+2k_{i} \end{array}$$

As shown in Figure 10, the product of the cosine of the slope angles and the correction lengths $k_{i}$ and $k_{e}$ would be the accurate adjustment. Due to the fact that Almen and Laszlo [39] use the small angle approximation and the integration over the area of the cross-section uses the diameters as limits, this is neglected in the second shape adjustment. Since the limits of the second integral uses the thickness *t*, the second adjustment only affects the diameters and it has no influence on the adjusted total height ( $l_{0}^{\prime \prime }=l_{0}^{\prime }$ ).

When applying this approach to other characteristic calculation methods, the choice of whether to apply these simplifications or not, has to be made individually for those other methods, based on the use of the small angle approximation and the limits in the calculation of resistance to deformation. In three of the four methods used in Section 6, this simplification was applicable due to small angle approximation in the definition of the integration limits in the original formulas. In the adjustment of the Kobelev-formula, the cosine noted above must be considered (see Appendix B).

The correction lengths $k_{i}$ and $k_{e}$ used in the second shape adjustment are defined by comparing the outer and inner part of a rectangular cross-section with a cross-section with rounded edges. To simplify this, two averaged edge radii, $r_{e}$ and $r_{i}$ , are introduced, which are defined so that the area of the cross-section remains constant. Equation (44) shows the balance of area differences to a cross-section with sharp edges to define $r_{i}$ :

(44) $$\begin{array}{cc} \left(1-\frac{\pi }{4}\right)2r_{i}^{2} & =\left(1-\frac{\pi }{4}\right)\left(r_{I}^{2}+r_{\mathrm{II}}^{2}\right) \end{array}$$

This can be solved for $r_{i}$ . The average outer edge radius $r_{e}$ can be computed analogously:

(45) $$\begin{array}{cc} r_{i} & =\sqrt{\frac{r_{I}^{2}+r_{\mathrm{II}}^{2}}{2}} \end{array}$$

(46) $$\begin{array}{c} r_{e}=\sqrt{\frac{r_{\mathrm{III}}^{2}+r_{\mathrm{IV}}^{2}}{2}} \end{array}$$

With those average edge radii, $r_{e}$ and $r_{i}$ , the correction lengths $k_{i}$ and $k_{e}$ for the inner and outer diameter are defined such that the cross-sectional area remains unchanged.

(47) $$\begin{array}{cc} t\;(r_{i}-k_{i}) & =2\;\frac{\pi }{4}\;r_{i}^{2}+\left(t-2r_{i}\right)\;r_{i} \end{array}$$

This can be solved for the inner correction length $k_{i}$ . In this consideration the influence of the angles $\beta_{i}$ and $\beta_{e}$ is neglected. The outer correction length $k_{e}$ can be computed analogously:

(48) $$\begin{array}{cc} k_{i} & =\frac{1}{2t}\;\left(4-\pi \right)\;r_{i}^{2} \end{array}$$

(49) $$\begin{array}{cc} k_{e} & =\frac{1}{2t}\;\left(4-\pi \right)\;r_{e}^{2} \end{array}$$

The correction lengths $k_{i}$ and $k_{e}$ from Equations (48) and (49) indicate by which quantity the diameters need to be adjusted to keep the area of the cross-section the same, see Equations (42) and (43).

### 5.2. Applying the Method to Almen’s Formula

To apply the method described above and to compute the characteristic of a disc spring with non-rectangular cross-section and rounded edges, the adjusting method is applied to the formulas published in 1937 by Almen and Laszlo [39].

Almen and Laszlo based their calculation on the equality of an internal moment $M_{i}$ and an external moment $M_{e}$ [39]:

(50) $$\begin{array}{c} M_{e}=M_{i} \end{array}$$

The internal moment $M_{i}$ results from the stress caused by the rotation of the cross-section [39]. Its computation is not modified here except for the use of the adjusted diameters $D_{e}^{\prime \prime }$ , $D_{i}^{\prime \prime }$ and the adjusted total height $l_{0}^{\prime \prime }$ derived above.

The external moment on a sector dϑ is calculated as the product of the part of the force $F_{A,o}$ applied on this sector and the lever arm of the force application [39]. Almen and Laszlo simplify the lever arm by measuring the distance between radii of the inner and outer edge of the neutral surface. According to [84], the use of the measured diameters instead of those of the neutral surface in the formulas results in a better match with characteristics determined experimentally. This measurement of the diameters is also done in the standard DIN EN 16984 [36] and by Zheng et al. [41], and thus is used in the current work for Almen–Laszlo-, Curti–Orlando and Zheng-based computation. It is not used for the Kobelev-based computation [85] as the results couldn’t be improved using different diameter definitions.

On a rectangular cross-section, the external moment is computed by

(51) $$\begin{array}{cc} M_{e} & =F_{A,o}\;\frac{d\vartheta }{2\pi }\;\left(\left(\frac{D_{e}}{2}-\sin\left(\varphi \right)\;\frac{t}{2}\right)-\left(\frac{D_{i}}{2}+\sin\left(\varphi \right)\;\frac{t}{2}\right)\right)\\ & =F_{A,o}\;\frac{d\vartheta }{2\pi }\;\left(R-\sin(\varphi )\;t\right) \end{array}$$

using the difference in radius defined in Equation (3).

Almen and Laszlo use this formula with the small angle approximation in a simplified form [39]:

(52) $$\begin{array}{cc} M_{e} & =F_{A,o}\;\frac{d\vartheta }{2\pi }\;R \end{array}$$

In order to address the non-rectangular cross-section and the rounded edges, the lever arm is not simplified by the difference in radius but described by the deflection angle-dependent lever arm $V_{\psi }$ defined in Equation (34). The external moment is calculated with Equation (53), so the force $F_{A}$ differs from the one calculated with the original Almen formula, $F_{A,o}$ :

(53) $$\begin{array}{cc} M_{e} & =F_{A}\;\frac{d\vartheta }{2\pi }\;V_{\psi } \end{array}$$

Taking the above replacement of the diameters in the underlying Almen-method and the more accurate lever arm $V_{\psi }$ into account, the force $F_{A}$ at a given axial deflection *s* is computed according to the adjusted Almen formula, Equation (7) in [39] by:

(54) $$\begin{array}{cc} F_{A}= & \frac{4\;E\;s\;t}{\left(1-\mu^{2}\right)\;{D_{e}^{\prime \prime }}^{2}\;M^{\prime \prime }}\left(\left(h_{0,\mathrm{DIN}}^{\prime \prime }-s\right)\;\left(h_{0,\mathrm{DIN}}^{\prime \prime }-\frac{s}{2}\right)+t^{2}\right) \end{array}$$

(55) $$\begin{array}{cc} \frac{1}{M^{\prime \prime }}= & \left(\frac{\delta^{\prime \prime }+1}{\delta^{\prime \prime }-1}-\frac{2}{\ln(\delta^{\prime \prime })}\right)\;\pi \;{\left(\frac{\delta^{\prime \prime }}{\delta^{\prime \prime }-1}\right)}^{\;2} \end{array}$$

where the adjusted diameter ratio $\delta^{\prime \prime }$ and the adjusted cone height using the small angle approximation $h_{0,\mathrm{DIN}}^{\prime \prime }$ is defined analogously to [39] p. 311, and Equation (1), respectively:

(56) $$\begin{array}{cc} \delta^{\prime \prime } & =\frac{D_{e}^{\prime \prime }}{D_{i}^{\prime \prime }} \end{array}$$

(57) $$\begin{array}{cc} h_{0,\mathrm{DIN}}^{\prime \prime } & =l_{0}^{\prime \prime }-t \end{array}$$

The Equations (52) and (53) lead to the following formula to replace the lever arm $V_{\psi }$ in the Almen-equation to compute the force $F_{A,o}$ :

(58) $$\begin{array}{cc} F_{A} & =F_{A,o}\frac{R}{V_{\psi }} \end{array}$$

Again, the above replacement of the diameters in the underlying Almen-method can be used in Equation (58), so the computation of the force $F_{A}$ can simply be written as:

(59) $$\begin{array}{cc} F_{A} & =F_{A,o}\left(D_{e}^{\prime \prime },D_{i}^{\prime \prime },l_{0}^{\prime \prime }\right)\;\frac{R^{\prime \prime }}{V_{\psi }} \end{array}$$

with the adjusted difference in radius defined analogously to Equation (3):

(60) $$\begin{array}{cc} R^{\prime \prime } & =\frac{D_{e}^{\prime \prime }-D_{i}^{\prime \prime }}{2} \end{array}$$

Equation (54) as well as Equation (59) derived above show an exemplary application of the new method introduced in Section 5.1 to adopt other algorithms to a non-rectangular cross-section with rounded edges. To show the effect of the adjustments, the method is also applied to the algorithm by Curti and Orlando [40], as well as the one by Zhang [41], by replacing the force $F_{A,o}$ computed using the original Almen formula with the force $F_{C,o}$ or $F_{Z,o}$ computed using Curti’s or Zheng’s original formulas, respectively. The algorithm by Kobelev [85] is also adapted by using the force $F_{K,o}$ instead of $F_{A,o}$ in the above equations and replacing the adjusted difference in radius *R* by the lever arm $H_{r,K}$ used by Kobelev. The final formulas are written out in full in Appendix B.

## 6. Results and Discussion

The characteristics computed according to the equations derived above are compared to those of Almen–Laszlo, Curti–Orlando, Zheng and Kobelev as well as results of FE simulations by applying them to multiple cross-sectional geometries. For all springs, Young’s modulus *E* is defined as 206 G Pa and the Poisson’s ratio μ is set to 0.3, as prescribed in [35].

**Figure 11:**
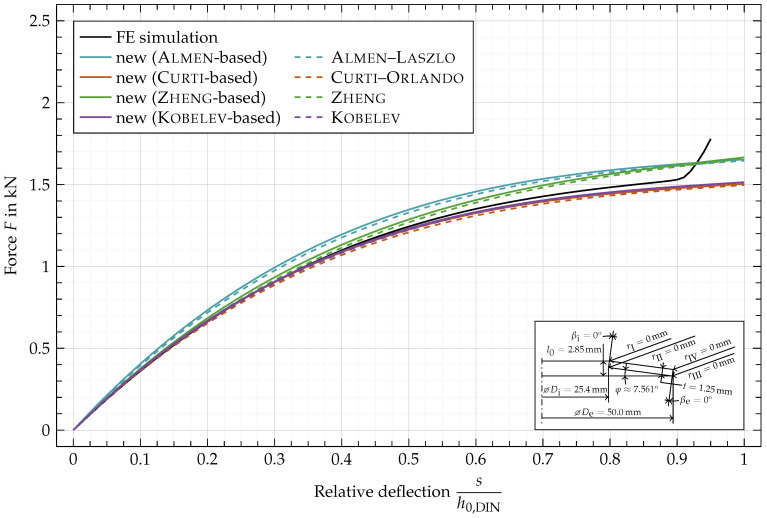
Characteristic comparison of a disc spring with $r_{I},r_{\mathrm{II}},r_{\mathrm{III}},r_{\mathrm{IV}}$ = 0 mm and $\beta_{i},\beta_{e}=0{}^{\circ}$ .

In Figure 11, the characteristic of disc spring EN 16983 —C 50 with a rectangular cross-section and sharp edges is shown. The deviation between the characteristic computed by the original formulas of Almen (Equation (A14), Figure 11, ---) and the adjusted ones based on Almen’s formulas (Equation (A7), Figure 11, —) are caused by the correction of the lever arm, which is simplified by Almen as the difference in radius *R* and thus constant during deflection in the original equations. In the new approach the deflection-dependent lever arm $V_{\psi }$ is often (see condition (38)) smaller than the difference in radius *R*. Due to the use of the slightly smaller deflection-dependent lever arm $V_{\psi }$ instead of the difference in radius *R*, the calculated forces are slightly larger, compared to the results of the original formulas. For large deflections, the lever arm elongates and gets closer to the difference in radius resulting in a decreasing relative difference between the adjusted and the original characteristics for rectangular cross-sections. The same applies to the original characteristics by Curti (Equation (A15), Figure 11, ---), Zheng (Equation (A16), Figure 11, ---) and Kobelv (Equation (A24), Figure 11, ---) and the adjusted characteristics based on those (Equations (A8)–(A10), Figure 11, —, — and —).

The increasing force of the characteristics obtained by FE simulation (Figure 11, —) starting at about $\frac{s}{h_{0,\mathrm{DIN}}}=0.9$ is caused by the setup of the simulation where the spring is compressed between two flat surfaces. Since this effect is neglected in the analytical approaches their characteristics don’t reflect this phenomenon.

It is immediately apparent that the characteristics based on Curti–Orlando and Kobelev better match the FE simulation. The characteristics based on Almen–Laszlo and Zheng overestimate the force, while Zheng still shows a good fit for small relative deflections below 0.15.

**Figure 12:**
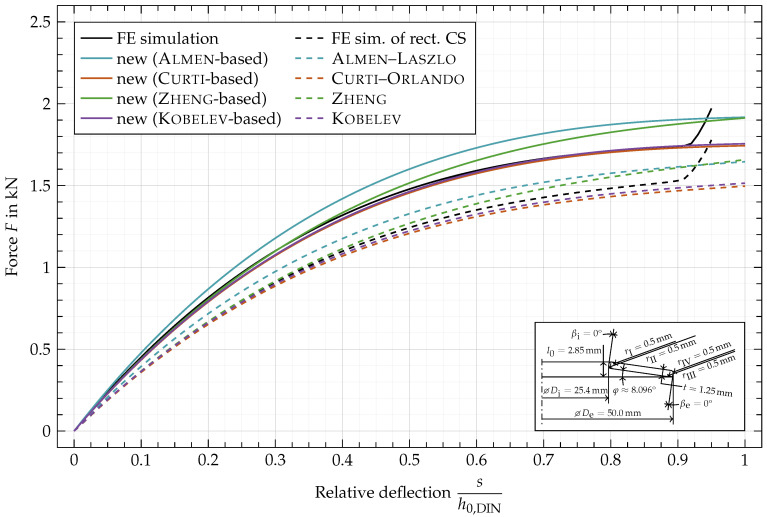
Characteristic comparison of a disc spring with $r_{I},r_{\mathrm{II}},r_{\mathrm{III}},r_{\mathrm{IV}}$ = 0.5 mm and $\beta_{i},\beta_{e}=0{}^{\circ}$ .

**Figure 13:**
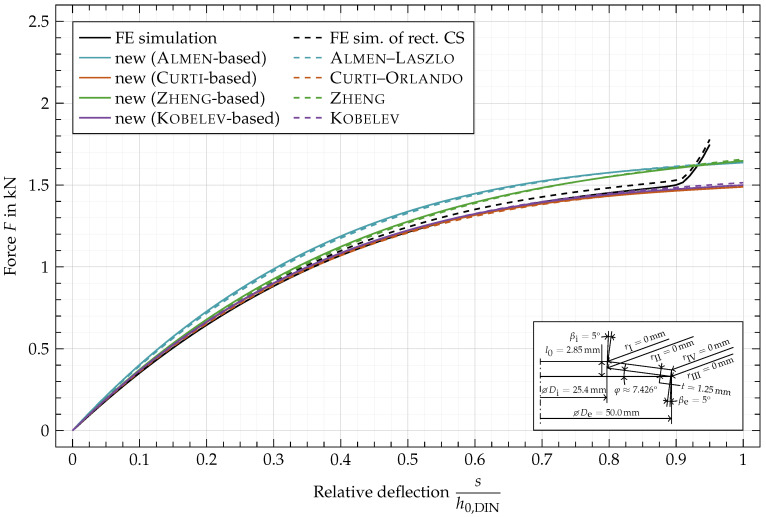
Characteristic comparison of a disc spring with $r_{I},r_{\mathrm{II}},r_{\mathrm{III}},r_{\mathrm{IV}}$ = 0 mm and $\beta_{i},\beta_{e}=5{}^{\circ}$ .

**Figure 14:**
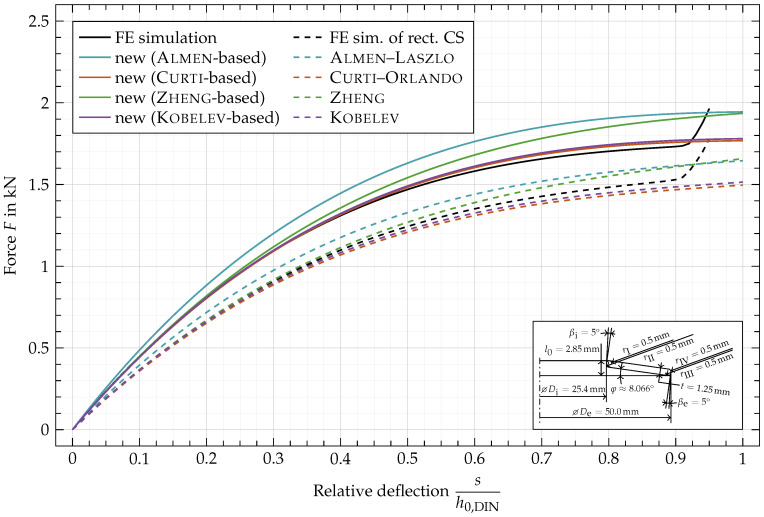
Characteristic comparison of a disc spring with $r_{I},r_{\mathrm{II}},r_{\mathrm{III}},r_{\mathrm{IV}}$ = 0.5 mm and $\beta_{i},\beta_{e}=5{}^{\circ}$ .

In Figure 12 and Figure 13, the same disc spring as in Figure 11 is shown but with rounded edges ( $r_{I},r_{\mathrm{II}},r_{\mathrm{III}},r_{\mathrm{IV}}$ = 0.5 mm, Figure 12) and non-rectangular cross-section ( $\beta_{i},\beta_{e}=5{}^{\circ}$ , Figure 13), respectively. In Figure 14, both variations of the cross-section are combined. To highlight the influence of the geometric variations, the FE simulation of the rectangular cross-section (Figure 11, —) is added as a black dashed line (Figure 12, ---) to the plots. Since the original formulas are based on a rectangular cross-sections, the dashed lines are equivalent to the ones of same color in Figure 11.

Figure 12 shows a substantial increase in the force with rounded edges with a 0.5 mm radius, i.e., two fifth of the sheet thickness each. The characteristics based on Curti–Orlando and Kobelev show a good fit to the FE simulation, while both show a slight underestimation around a relative deflection of 0.35. The characteristics based on Almen–Laszlo and Zheng et al. show the same divergence from the simulated characteristics as for a rectangular cross-section. This can be explained by the fact that the new approach is based on the original formulas and doesn’t account for the deviation of the characteristics computed with the original formulas from those computed by FE simulations.

In Figure 13, the angles of the inner and outer faces, $\beta_{i}$ and $\beta_{e}$ , are both set to 5°. This causes a small decrease in force, both in the FE simulation and in the computed characteristics. Both show smaller forces compared to the results from the rectangular cross-section in Figure 11. The difference between the two FE simulated characteristics is slightly larger than the difference between the analytically computed (colored, solid) ones from the two figures. This indicates that the effect from angled inner and outer faces is not completely covered by the analytical description derived in the current work.

A combination of the two cross-sectional variations from Figure 12 and Figure 13 is applied in one single disc spring geometry. The characteristics of the cross-section with both, radii of 0.5 mm and angles of the inner and outer face of 5°, are shown in Figure 14. It shows again the slight overestimation in force along with $\beta_{i}$ and $\beta_{e}$ at 5°, but the general matches between the characteristics based on Curti–Orlando and Kobelev are still good, especially for relative deflections below 0.5.

To compare the results quantitatively, the maximum relative error ( $\left|F_{i,o}-F_{\mathrm{FE}}\right|/F_{\mathrm{FE}}$ ) was used, where $F_{\mathrm{FE}}$ is the force computed by FE simulation and $F_{i}$ is the force computed by the new method based on formulas by different authors abbreviated as *i*. Relative deflections above 0.8 were not included in the analysis since the FE simulation shows a progressively increasing force for large deflections, as discussed above. The results for the Curti–Orlando and Kobelev based characteristics are shown in Table 1. At less than 8, the maximum relative errors of the characteristics of disc springs with geometric variations are in the same order of magnitude as those of springs with geometries with a rectangular cross-section and sharp edges.

In Appendix C, characteristics and maximum relative errors for more geometric variations are shown. The correction made by the adjustments is not large enough to reflect the changes in the simulated characteristic caused by negative angles $\beta_{e}$ and $\beta_{i}$ . For negative angles larger in magnitude (−10°), this divergence increases over-proportionally and leads to a better fit of the FE simulated characteristics with the results of Zheng, which is likely due to the compensation of errors. Large angles (10°) cause the characteristics based on Curti and Kobelev to overestimate the force over a wide range of relative deflections (here, 0.2 to 0.8).

The new analytical method is a computationally inexpensive alternative to FE simulations of complex cross-sectional disc spring geometries. The new approach shows good characteristic adjustments while depending on an accurate characteristic computation method for disc springs with rectangular cross-section with sharp edges. The only weaknesses not attributable to the original formula were found with large angles of the inner and outer face. While those cases cannot be generally discounted, they are unlikely to occur. For large angles, the geometry quantities should be interpreted as outlined at the end of Section 2.

The best results were obtained by using the formulas of Curti–Orlando or Kobelev as base formulas, with the formula of Kobelev showing marginally better results. The method to adapt an existing formula to special cross-sections outlined in the current work may be adapted to other existing or future methods.

## 7. Conclusions

This paper presents the following findings:

1. A set of formulas were introduced to describe the geometric relations of the cross-section of a disc spring with non-rectangular cross-section and rounded edges. They enable the formulation of a more accurate way to compute the lever arm, taking its elongation during deflection into account.
2. A method adapting characteristic calculation formulas and algorithms to non-rectangular cross-sections with rounded edges was outlined. This new adaption method can be applied to different traditional characteristic calculation algorithms designed for disc springs with a rectangular cross-section. The method was exemplarily applied to the algorithms of Almen–Laszlo, Curti–Orlando, Zheng and Kobelev to show its applicability.
3. The adjusted formulas were used to calculate characteristics of different disc springs. The results were compared to those of the original formulas and those obtained by FE simulations. Provided that the original characteristics approximate those of rectangular cross-sectional disc springs well, the adjusted characteristics based on them fit well for most investigated geometries. Based on the results of exemplarily computed characteristics, we recommend the use of the proposed method, utilizing formulas introduced by Curti–Orlando or Kobelev. The relative errors made with the new method are in the same order of magnitude as those of geometries with rectangular cross-section.

## Figures and Tables

**Table 1:** Maximum relative errors of the adjusted characteristics.

Curti–Orlando Based			Kobelev Based	
$\beta_{i},\beta_{e}$	$r_{I},\ldots ,r_{\mathrm{IV}}=0\;\mathrm{mm}$	$r_{I},\ldots ,r_{\mathrm{IV}}=0.5\;\mathrm{mm}$	$r_{I},\ldots ,r_{\mathrm{IV}}=0\;\mathrm{mm}$	$r_{I},\ldots ,r_{\mathrm{IV}}=0.5\;\mathrm{mm}$
0°	5.09%	4.22%	5.58%	3.69%
5°	7.36%	1.73%	7.88%	2.35%

**Figure A1:**
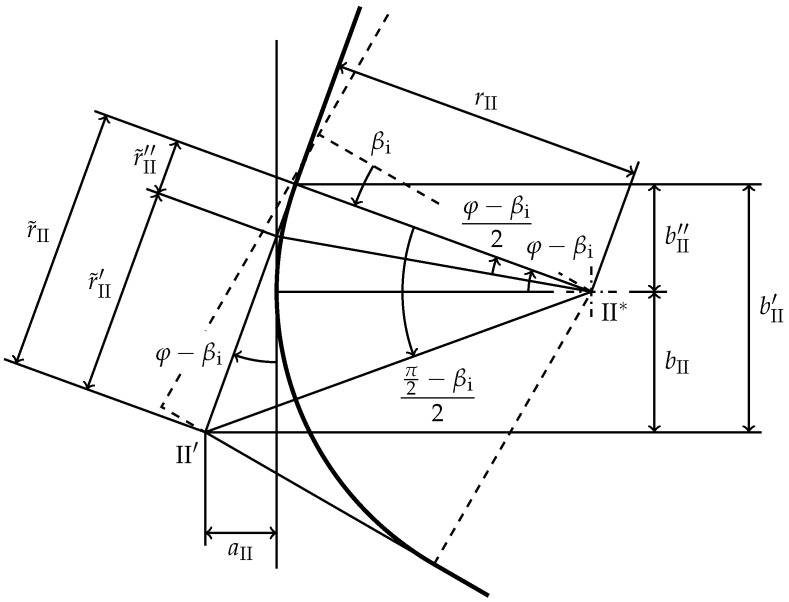
Geometry of edge II to calculate $a_{\mathrm{II}}$ and $b_{\mathrm{II}}$ ( $\beta_{i}>0$ ).

**Figure A2:**
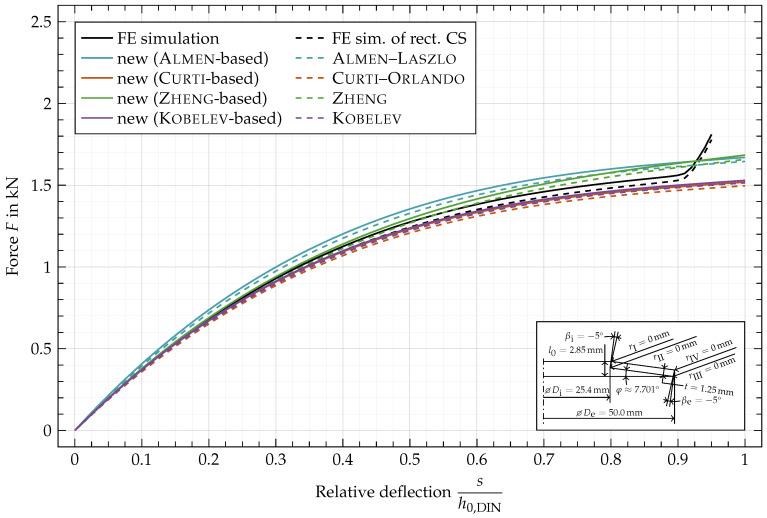
Characteristic comparison of a disc spring with $r_{I},r_{\mathrm{II}},r_{\mathrm{III}},r_{\mathrm{IV}}=0mm$ and $\beta_{i},\beta_{e}=-5{}^{\circ}$ .

**Figure A3:**
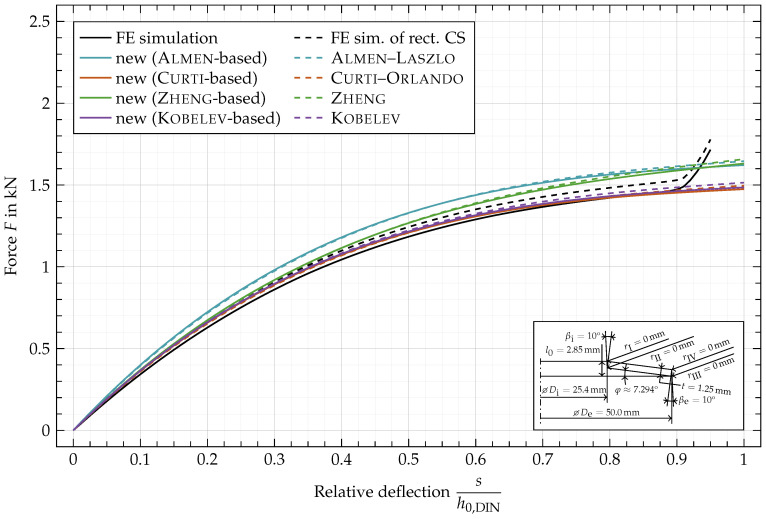
Characteristic comparison of a disc spring with $r_{I},r_{\mathrm{II}},r_{\mathrm{III}},r_{\mathrm{IV}}=0mm$ and $\beta_{i},\beta_{e}=10{}^{\circ}$ .

**Figure A4:**
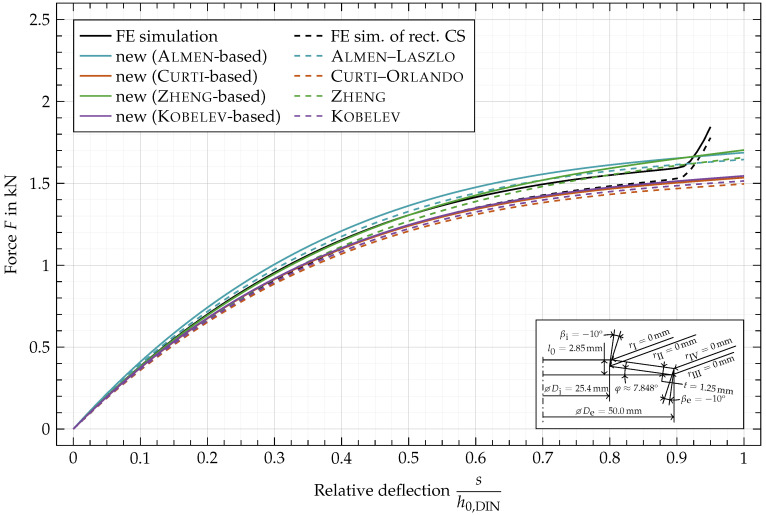
Characteristic comparison of a disc spring with $r_{I},r_{\mathrm{II}},r_{\mathrm{III}},r_{\mathrm{IV}}=0mm$ and $\beta_{i},\beta_{e}=-10{}^{\circ}$ .

**Figure A5:**
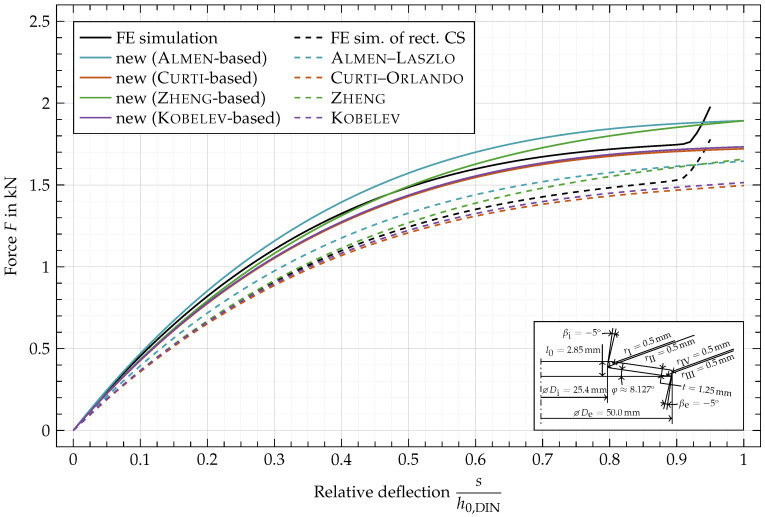
Characteristic comparison of a disc spring with $r_{I},r_{\mathrm{II}},r_{\mathrm{III}},r_{\mathrm{IV}}=0.5mm$ and $\beta_{i},\beta_{e}=-5{}^{\circ}$ .

**Figure A6:**
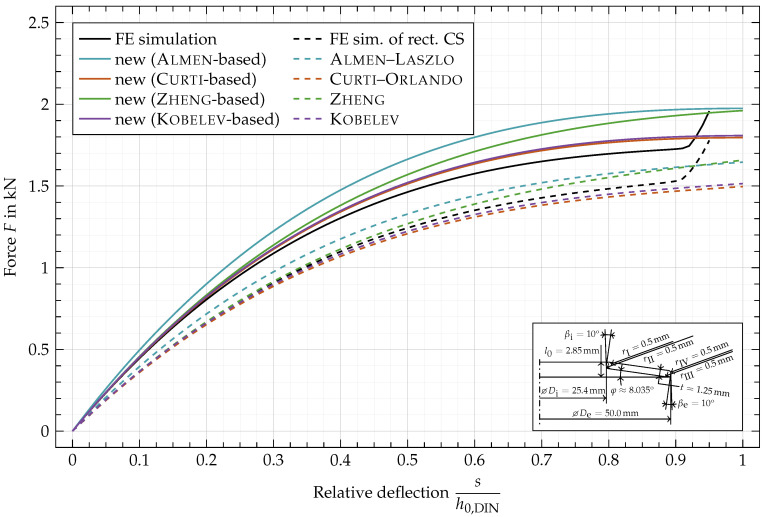
Characteristic comparison of a disc spring with $r_{I},r_{\mathrm{II}},r_{\mathrm{III}},r_{\mathrm{IV}}=0.5mm$ and $\beta_{i},\beta_{e}=10{}^{\circ}$ .

**Figure A7:**
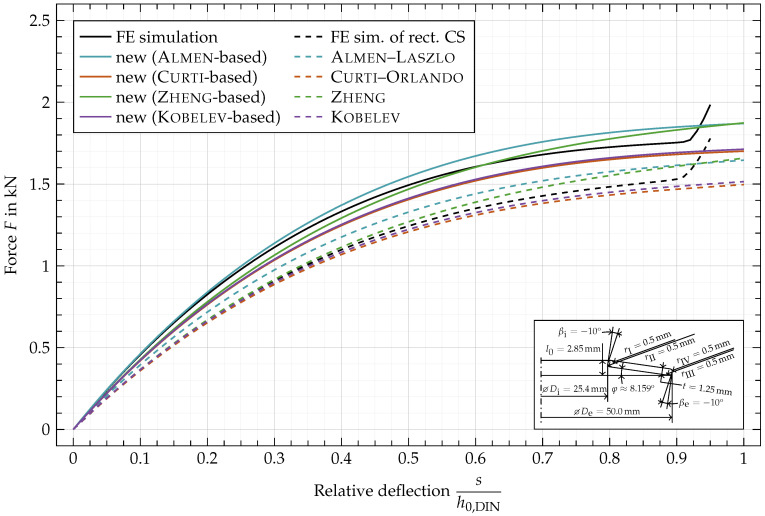
Characteristic comparison of a disc spring with $r_{I},r_{\mathrm{II}},r_{\mathrm{III}},r_{\mathrm{IV}}=0.5mm$ and $\beta_{i},\beta_{e}=-10{}^{\circ}$ .

**Figure A8:**
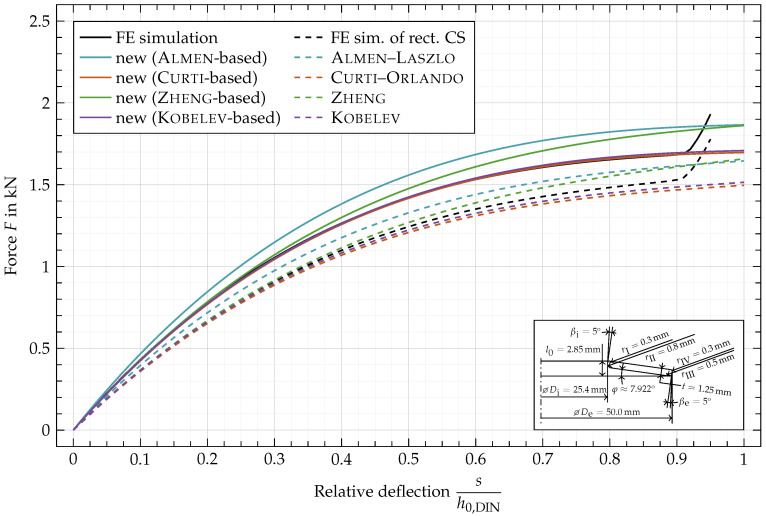
Characteristic comparison of a disc spring with $r_{I}=0.3mm,r_{\mathrm{II}}=0.8mm,r_{\mathrm{III}}=0.5mm,r_{\mathrm{IV}}=0.3mm$ and $\beta_{i},\beta_{e}=5{}^{\circ}$ .

## Appendix A. Derivation of $a_{\mathrm{II}}$ through $a_{\mathrm{IV}}$ and $b_{\mathrm{II}}$ through $b_{\mathrm{IV}}$

Analogously to $a_{I}$ and $b_{I}$ , $a_{\mathrm{II}}$ and $b_{\mathrm{II}}$ can be obtained from Figure A1.

The following equations emerge from the relations on edge II :

(A1)
$$\begin{array}{cc} a_{\mathrm{II}} & =\sin\left(\varphi -\beta_{i}\right)\left(\;\tan\left(\frac{\frac{\pi }{2}-\beta_{i}}{2}\right)-\tan\left(\frac{\varphi -\beta_{i}}{2}\right)\;\right)r_{\mathrm{II}} \end{array}$$

(A2)
$$\begin{array}{cc} b_{\mathrm{II}} & =\cos\left(\varphi -\beta_{i}\right)\left(\;\tan\left(\frac{\frac{\pi }{2}-\beta_{i}}{2}\right)-\tan\left(\varphi -\beta_{i}\right)\right)r_{\mathrm{II}} \end{array}$$

As can be seen by comparing Equations (A1) and (A2) with Equations (17) and (21), respectively, the above equations can be obtained by replacing φ by $\varphi -\beta_{i}$ and $\frac{\pi }{2}+\beta_{i}$ by $\frac{\pi }{2}-\beta_{i}$ as well as substituting the radius $r_{I}$ by $r_{\mathrm{II}}$ in the equations.

Essentially, the equations of the remaining two edges III and IV can be obtained by simply rotating Figure 7, Figure 8 and Figure A1 by 180° and replacing $\beta_{i}$ by $\beta_{e}$ , $r_{I}$ by $r_{\mathrm{III}}$ and $r_{\mathrm{II}}$ by $r_{\mathrm{IV}}$ .

In this way the following equations can be obtained to compute $a_{\mathrm{III}}$ , $b_{\mathrm{III}}$ :

(A3)
$$\begin{array}{cc} a_{\mathrm{III}} & =\sin\left(\varphi \right)\left(\;\tan\left(\frac{\frac{\pi }{2}+\beta_{e}}{2}\right)-\tan\left(\frac{\varphi }{2}\right)\;\right)r_{\mathrm{III}} \end{array}$$

(A4)
$$\begin{array}{cc} b_{\mathrm{III}} & =\cos\left(\varphi \right)\left(\;\tan\left(\frac{\frac{\pi }{2}+\beta_{e}}{2}\right)-\tan\left(\varphi \right)\right)r_{\mathrm{III}} \end{array}$$

as well as $a_{\mathrm{IV}}$ and $b_{\mathrm{IV}}$ :

(A5)
$$\begin{array}{cc} a_{\mathrm{IV}} & =\sin\left(\varphi -\beta_{e}\right)\left(\;\tan\left(\frac{\frac{\pi }{2}-\beta_{e}}{2}\right)-\tan\left(\frac{\varphi -\beta_{e}}{2}\right)\;\right)\;r_{\mathrm{IV}} \end{array}$$

(A6)
$$\begin{array}{cc} b_{\mathrm{IV}} & =\cos\left(\varphi -\beta_{e}\right)\left(\;\tan\left(\frac{\frac{\pi }{2}-\beta_{e}}{2}\right)-\tan\left(\varphi -\beta_{e}\right)\right)r_{\mathrm{IV}} \end{array}$$

## Appendix B. Full Equations

Below the adapted formulas based on Almen and Laszlo, Curti and Orlando, Zheng et al. as well as Kobelev are written out.

(A7)
$$\begin{array}{cc} F_{A} & =F_{A,o}\left(D_{e}^{\prime \prime },D_{i}^{\prime \prime },l_{0}^{\prime \prime }\right)\;\frac{R^{\prime \prime }}{V_{\psi }} \end{array}$$

(A8)
$$\begin{array}{cc} F_{C} & =F_{C,o}\left(D_{e}^{\prime \prime },D_{i}^{\prime \prime },l_{0}^{\prime \prime }\right)\;\frac{R^{\prime \prime }}{V_{\psi }} \end{array}$$

(A9)
$$\begin{array}{cc} F_{Z} & =F_{Z,o}\left(D_{e}^{\prime \prime },D_{i}^{\prime \prime },l_{0}^{\prime \prime }\right)\;\frac{R^{\prime \prime }}{V_{\psi }} \end{array}$$

(A10)
$$\begin{array}{cc} F_{K} & =F_{K,o}\left(D_{e}^{\prime \prime },D_{i}^{\prime \prime },l_{0}^{\prime \prime }\right)\;\frac{H_{r,K}}{V_{\psi }} \end{array}$$

(A11)
$$\begin{array}{cc} \mathrm{where}\;H_{r,K} & =r_{i,K}\;\frac{\cos(\psi_{K})}{\cos(\alpha_{K})}\;\left(\Delta_{K}-1\right) \end{array}$$

where the argument $\left(D_{e}^{\prime \prime },D_{i}^{\prime \prime },l_{0}^{\prime \prime }\right)$ denotates that the original forces need to be computed using $D_{e}^{\prime \prime }$ , $D_{i}^{\prime \prime }$ and $l_{0}^{\prime \prime }$ instead of $D_{e}$ , $D_{i}$ and $l_{0}$ , in quantities defined using them or directly in the formulas below, e.g., $h_{0,\mathrm{DIN}}$ is replaced by $h_{0,\mathrm{DIN}}^{\prime \prime }=l_{0}^{\prime \prime }-t$ . Note the different calculation of $D_{e}^{\prime \prime }$ and $D_{i}^{\prime \prime }$ discussed in Section 5.1 for the Kobelev-based adjustment, in which the following definition should be used instead of Equations (42) and (43):

(A12)
$$D_{e}^{\prime \prime }=D_{e}^{\prime }-2k_{e}\cos(\varphi )$$

(A13)
$$D_{i}^{\prime \prime }=D_{i}^{\prime }+2k_{i}\cos(\varphi )$$

The original forces of Almen–Laszlo [39] $F_{A,o}$ , Curti–Orlando [40] $F_{C,o}$ and Zheng [41] $F_{Z,o}$ are calculated as follows:

(A14)
$$\begin{array}{cc} F_{A,o} & =\frac{4E}{1-\mu^{2}}\frac{t^{3}\;s}{D_{e}^{2}}\frac{1}{M_{A}}\;\cdot \;\left(1+\left(\frac{h_{0,\mathrm{DIN}}}{t}-\frac{s}{t}\right)\left(\frac{h_{0,\mathrm{DIN}}}{t}-\frac{s}{2t}\right)\;\right) \end{array}$$

(A15)
$$\begin{array}{cc} F_{C,o} & =\frac{4E}{1-\mu^{2}}\frac{t^{3}\;s}{D_{e}^{2}}\frac{1}{M_{C}}\;\cdot \;\left(1+\left(\frac{h_{0,\mathrm{DIN}}}{t}-\frac{s}{t}\right)\left(\frac{h_{0,\mathrm{DIN}}}{t}-\frac{s}{2t}\right)\;\right) \end{array}$$

(A16)
$$\begin{array}{cc} F_{Z,o} & =\frac{4E}{1-\mu^{2}}\;\pi \;\alpha_{1,Z}\;\cdot \;\left(1+\alpha_{0,Z}\;\cdot \;\left(\frac{h_{0,\mathrm{DIN}}}{t}-\frac{s}{t}\right)\left(\frac{h_{0,\mathrm{DIN}}}{t}-\frac{s}{2\;t}\right)\;\right) \end{array}$$

(A17)
$$\begin{array}{cc} \mathrm{where}\;\frac{1}{M_{A}} & =\left(\frac{\delta +1}{\delta -1}-\frac{2}{\ln\left(\delta \right)}\right)\;\pi \;{\left(\frac{\delta }{\delta -1}\right)}^{\;2} \end{array}$$

(A18)
$$\begin{array}{cc} \frac{1}{M_{C}} & =\left(1-\mu^{2}\right)\frac{2\;\pi }{1-\mu }\frac{\delta^{2}}{{\left(\delta -1\right)}^{3}}\;\cdot \;\left(\frac{1+\delta }{2}+\frac{\mu }{1+\mu }+\frac{\delta^{\mu +1}-1}{1-\delta^{\mu }}\right) \end{array}$$

(A19)
$$\begin{array}{cc} \alpha_{0,Z} & =\left(1-\mu^{2}\right)\frac{6}{{\left(\delta -1\right)}^{2}\ln\left(\delta \right)}\;\cdot \;\left(\frac{1}{4}\left(\delta^{2}-1\right)-\frac{\delta^{2}}{\delta^{2}-1}\ln{\left(\delta \right)}^{2}\right) \end{array}$$

(A20)
$$\begin{array}{cc} \alpha_{1,Z} & =\frac{1}{6}{\left(\frac{\delta }{\delta -1}\right)}^{\;2}\ln\left(\delta \right) \end{array}$$

To compute the force $F_{K,o}$ with the disc spring equations by Kobelev [85], the following algorithm was used: Since the geometry definition by Kobelev is based on the radii $r_{i,K}$ , $r_{e,K}$ at the middle of the inner and outer face, these quantities must first be computed. This is done iteratively with the following three equations until convergence is achieved:

(A21)
$$\begin{array}{cc} \alpha_{K} & =\arctan\left(\frac{l_{0}-t}{r_{e,K}-r_{i,K}}\right) \end{array}$$

(A22)
$$\begin{array}{cc} r_{i,K} & =\frac{D_{i}}{2}+\sin\left(\alpha_{K}\right)\times \frac{t}{2} \end{array}$$

(A23)
$$\begin{array}{cc} r_{e,K} & =\frac{D_{e}}{2}-\sin\left(\alpha_{K}\right)\times \frac{t}{2} \end{array}$$

According to Equations (4.33) through (4.35) in [85], those quantities can then be used to compute the force:

(A24)
$$\begin{array}{cc} F_{K,o} & =\pi \;E\;r_{i,K}^{2}\frac{F_{e,K}\;\mu_{K}+F_{f,K}\;\mu_{K}^{3}}{\cos\left(\psi_{K}\right)} \end{array}$$

(A25)
$$\begin{array}{cc} F_{e,K} & =\frac{2\left(1-\Delta_{K}\right)+\left(1+\Delta_{K}\right)\;\ln\left(\Delta_{K}\right)}{\ln(\Delta_{K})}\;\cdot \;\frac{\cos\left(\psi_{K}\right)-\cos\left(\alpha_{K}\right)}{\cos^{2}\left(\alpha_{K}\right)/\sin\left(\psi_{K}\right)} \end{array}$$

(A26)
$$\begin{array}{cc} F_{f,K} & =\frac{\ln\left(\Delta_{K}\right)}{6\;\left(\Delta_{K}-1\right)}\;\cdot \;(\sin\left(\alpha_{K}\right)-\sin\left(\psi_{K}\right))\;\cos\left(\psi_{K}\right) \end{array}$$

where

(A27)
$$\begin{array}{cc} \mu_{K}= & \frac{t}{r_{i,K}} \end{array}$$

(A28)
$$\begin{array}{cc} \psi_{K}= & \arcsin\left(\frac{H_{K}}{x_{i,K}-x_{e,K}}\right) \end{array}$$

(A29)
$$\begin{array}{cc} H_{K}= & l_{0}-t-s \end{array}$$

(A30)
$$\begin{array}{cc} x_{i,K}= & \frac{c_{i,K}-r_{i,K}}{\cos\left(\alpha_{K}\right)} \end{array}$$

(A31)
$$\begin{array}{cc} x_{e,K}= & \frac{c_{i,K}-r_{e,K}}{\cos\left(\alpha_{K}\right)} \end{array}$$

(A32)
$$\begin{array}{cc} c_{i,K}= & \frac{\Delta_{K}-1}{\ln\left(\Delta_{K}\right)}\times r_{i,K} \end{array}$$

(A33)
$$\begin{array}{cc} \Delta_{K}= & \frac{r_{e,K}}{r_{i,K}}, \end{array}$$

according to Equations (4.4), (4.13), (4.14), (4.30), (4.31) and Equation on p. 100 in [85].

## Appendix C. Force vs. Deflection Curves for Additional Geometric Variations

Below, more examples of geometric variations are shown. The maximum relative errors are shown in Table A1. The base geometry with a rectangular cross-section and sharp edges can be found in Figure 11.

**Table A1:** Maximum relative errors of the adjusted characteristics for additional geometric variations.

Curti–Orlando Based			Kobelev Based	
$\beta_{i},\beta_{e}$	$r_{I},\ldots ,r_{\mathrm{IV}}=0\;\mathrm{mm}$	$r_{I},\ldots ,r_{\mathrm{IV}}=0.5\;\mathrm{mm}$	$r_{I},\ldots ,r_{\mathrm{IV}}=0\;\mathrm{mm}$	$r_{I},\ldots ,r_{\mathrm{IV}}=0.5\;\mathrm{mm}$
10°	9.64%	4.06%	10.18%	4.68%
5°	7.36%	1.73%	17.88%	2.35%
0°	5.09%	4.22%	15.58%	3.69%
−5°	3.98%	6.37%	13.37%	5.87%
−10°	5.41%	8.47%	14.82%	8.00%
$r_{I}=0.3\;\mathrm{mm},r_{\mathrm{II}}=0.8\;\mathrm{mm},r_{\mathrm{III}}=0.5\;\mathrm{mm},r_{\mathrm{IV}}=0.3\;\mathrm{mm}$				
5°	1.97%		1.43%	
